# Enhanced Vision-Based Quality Inspection: A Multiview Artificial Intelligence Framework for Defect Detection

**DOI:** 10.3390/s25061703

**Published:** 2025-03-10

**Authors:** Geethika Bhavanasi, Davy Neven, Manuel Arteaga, Sina Ditzel, Sam Dehaeck, Abdellatif Bey-Temsamani

**Affiliations:** Flanders Make, Oude Diestersebaan 133, 3920 Lommel, Belgium

**Keywords:** defect detection, deep learning, early fusion, late fusion, active vision, segmentation, multiview analysis

## Abstract

Automated defect detection is a critical component of modern industrial quality control. However, it is particularly difficult to identify subtle defects such as scratches on metallic surfaces. Therefore, this paper investigates the effectiveness of multiview deep learning approaches for improved defect detection by implementing and comparing early and late fusion methodologies. We propose MV-UNet, a novel early fusion architecture that aligns and aggregates multiview features using a transformation block to enhance detection accuracy. To evaluate performance, we conduct our experiments on a recorded metallic plates dataset, comparing the traditional single-view inspection to both late and early fusion methods. Our results demonstrate that both the early and late fusion methods improve detection accuracy over the mono-view baseline, with our MV-UNet achieving the hightest F1-score (0.942). Additionally, we introduce adapted precision–recall metrics designed for segmentation-based defect detection, addressing the limitations of traditional IoU-based evaluations. These tailored metrics more accurately reflect defect localization performance, particularly for thin, elongated scratches. Our findings highlight the advantages of early fusion for industrial defect detection, providing a robust and scalable approach to multiview analysis.

## 1. Introduction

The integration of computer vision and deep learning has transformed defect detection in industrial quality control, reducing manual labor, minimizing errors, and accelerating inspection processes [1]. Despite these advancements, detecting subtle and complex defects, such as scratches on industrial components, remains a critical challenge. These fine defects are often difficult to identify using static or single-view systems due to their dependence on viewing angles and surface variations. Ensuring thorough inspection for such defects requires multiview analysis, which captures objects from multiple perspectives to enhance detection accuracy and robustness [2].

Traditional defect detection systems are limited in their ability to adapt to the dynamic demands of industrial environments, where factors such as uneven surfaces, variable lighting, and defect intricacies pose significant challenges. Addressing these limitations necessitates an innovative combination of artificial intelligence techniques and adaptive imaging systems to maximize detection precision and scalability.

Multiview defect detection mimics the human inspection process by examining objects from various perspectives, allowing for a more comprehensive understanding of surface defects. By integrating observations from different angles, multiview systems can effectively capture subtle, irregular, or occluded defects that may remain undetected from a single viewpoint. This process ensures a holistic assessment of the object’s surface, enhancing the robustness and reliability of defect detection systems. However, effectively combining information from multiple views requires advanced fusion techniques to reason jointly about the data, ensuring accurate and consistent predictions across perspectives.

Given the challenges posed by irregular and subtle defects, such as long and thin scratches, traditional box-based methods like instance segmentation are often inadequate. Semantic segmentation, which provides pixel-level classification, is better suited for detecting such defects, making it the foundation of our proposed defect detection framework [3].

This paper introduces a novel artificial intelligence (AI)-driven defect detection framework that leverages multiview image analysis and advanced fusion techniques. At the core of this framework are two newly developed fusion networks—early fusion and late fusion—based on the Multiview UNet (MV-UNet) architecture. These networks effectively integrate multiview information to address the limitations of single-view approaches, significantly improving accuracy in detecting subtle defects, such as scratches on industrial plates. The fusion networks build on a baseline semantic segmentation model, enhancing their capability to localize and classify defects with greater precision.

To enable multiview analysis, the framework incorporates an active vision setup. This system employs a robotic arm to dynamically adjust camera viewpoints, capturing images from various angles for comprehensive visual coverage. By ensuring that defects are visible from multiple perspectives, this setup enhances the framework’s robustness in detecting even the smallest or most complex defects [4].

Another key component of the framework is an auto-annotation tool, designed to streamline the labor-intensive process of annotating training data. Leveraging the active vision system’s ability to capture multiview data, the tool automates the generation of annotations by projecting manually labeled points onto other views, reducing manual effort and ensuring consistency across datasets. This approach dramatically accelerates the preparation of high-quality datasets, which are critical for training fusion networks.

**Figure 1:**
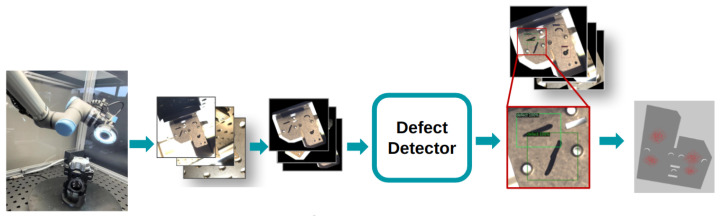
The main artificial intelligence (AI) defect detection steps, from image capture to prediction and fusion. The steps involve the following: image recording, capturing recorded image’s computer-aided design (CAD) + Camera position, image preprocessing, defect detecting, predictions (boxes, labels, scores, and masks), and fusion.

The proposed AI framework is implemented through a structured pipeline that integrates the following key stages (Figure 1):

1. Data Collection: A robotic system positions a camera to capture images from various viewpoints, ensuring complete coverage of the component under inspection.
2. Annotation: The captured images are annotated to label defects, using an auto-annotation tool to efficiently generate consistent, high-quality labels.
3. Defect Detection: Advanced deep learning models, including Feature Pyramid Network (FPN) [5] with Residual Neural Network (ResNet)34 [6], are used for detecting defects. The early and late fusion networks further enhance the detection accuracy by leveraging multiview analysis.
4. Prediction Refinement: The outputs of the fusion networks are processed to produce the final predictions, ensuring the precise localization and identification of defects.

Additionally, a new evaluation metric is introduced to address the limitations of standard metrics, providing a more reliable and detailed assessment of defect detection performance. This novel metric, tailored specifically for industrial defect detection tasks, ensures accurate comparisons between models and highlights the superior capabilities of the proposed fusion networks.

The key contributions of this paper are as follows:

1. Novel Fusion Networks: Early and late fusion networks, developed under the MV-UNet architecture, integrate multiview information to significantly improve detection accuracy, particularly for small defects like scratches.
2. Novel Evaluation Metric: A custom evaluation metric is introduced, tailored to defect detection tasks, addressing the limitations of standard metrics and providing more reliable performance assessments.
3. AI Framework for Multiview Analysis: Demonstration of a complete AI pipeline that integrates multiview image analysis, annotation, and prediction refinement for industrial defect detection.

While the active vision setup and auto-annotation tool are integral to the framework, the main focus of the paper is on the fusion networks, their implementation, and their evaluation in improving the defect detection accuracy.

## 2. Related Work

The application of AI in defect detection has undergone significant advancements, particularly with the adoption of deep learning and convolutional neural networks (CNNs) [7]. Early efforts demonstrated the potential of AI-based defect detection systems using basic neural network architectures. Although these methods automated certain aspects of quality control, their single-view nature limited their ability to detect subtle or complex defects, such as scratches, which often depend on viewing angles and lighting conditions.

To overcome these limitations, multiview inspection techniques emerged as a promising approach. Steitz et al. [8] applied CNNs to multiview datasets, showing that analyzing an object from multiple angles could improve detection accuracy. Similarly, works such as those of Li et al. [9] emphasized the importance of capturing diverse perspectives for inspecting textured surfaces or highly reflective materials. However, these methods often relied on fixed camera setups, which reduced their adaptability to real-world industrial environments with diverse inspection needs.

Dynamic viewpoint adjustment further advanced the field as demonstrated by Wu et al. [2], who developed a multiview learning approach for subsurface defect detection in composite products using thermographic data analysis. This approach ensured better coverage of the components inspected but focused primarily on hardware flexibility rather than optimizing AI-driven detection processes. Although our work incorporates a dynamic viewpoint optimization setup for capturing multiview data, the details are beyond the scope of this paper and will be addressed in future research.

Additionally, robotic inspection systems have been explored for defect detection in structured environments. Loupos et al. [10] developed an autonomous robotic system for tunnel structural inspection, integrating advanced sensors and vision-based algorithms for defect detection. Similarly, Stentoumis et al. [11] proposed a robotic inspector for tunnel assessment, leveraging deep learning techniques to identify surface defects. These approaches demonstrate the potential of robotic-assisted inspection, aligning with our active vision setup for defect detection.

The integration of multiview predictions through fusion networks has been another area of interest. Jiang et al. [12] proposed a fusion-based approach to aggregate information from multiple views, improving the accuracy of defect detection. However, their work relied primarily on view-level weight strategies, which did not fully exploit feature-level interactions between views. Earlier works explored feature-level fusion but did not apply these techniques to industrial defect detection scenarios. Our approach extends this line of research by introducing MV-UNet, a novel architecture that combines early and late fusion strategies tailored to subtle defect detection. This hybrid approach leverages both feature-level and decision-level integration to achieve superior performance, particularly for fine-grained defects like scratches.

More recent approaches, such as those of Chen et al. [3], explored hybrid fusion techniques for multiview object recognition, showcasing the potential of combining early and late fusion strategies to improve robustness. However, these methods were rarely tested on subtle defects, such as scratches, where fine-grained details are critical for accurate detection.

Evaluation metrics are a critical component of defect detection research. While standard metrics like Mean Average Precision (mAP) and Intersection over Union (IoU) are widely used, they often fail to capture the nuances of fine-grained defect detection. The work by Islam et al. [1] introduced metrics tailored for high-resolution defect detection, emphasizing pixel-level precision. These advancements highlight the need for task-specific metrics, which our work addresses through the development of a novel evaluation metric tailored to subtle defect detection tasks.

Building on these advancements, our research introduces a comprehensive AI-driven defect detection framework that integrates MV-UNet-based multiview prediction fusion and a novel evaluation metric tailored for subtle defects. In contrast to prior works that emphasized static multiview setups or general-purpose metrics, our framework combines advanced AI techniques with adaptive imaging systems to achieve greater precision and scalability. By focusing on these AI-driven contributions, our system addresses critical gaps in multiview defect detection, demonstrating significant improvements in identifying subtle defects in industrial quality control applications.

While multiview and robotic inspection approaches enhance defect detection, real-world industrial applications present additional challenges. Lighting variations, surface reflectivity, occlusions, and limited viewing angles may require additional viewpoints for accurate defect identification. Additionally, balancing detection precision with real-time processing efficiency is crucial for deployment in industrial settings. Robotic movement constraints further impact viewpoint selection, necessitating optimization for full coverage. Our approach integrates multiview analysis and fusion techniques to address these challenges, ensuring robustness and efficiency in defect detection.

## 3. Methodology

### 3.1. Overview of AI Defect Detection

**Figure 2:**
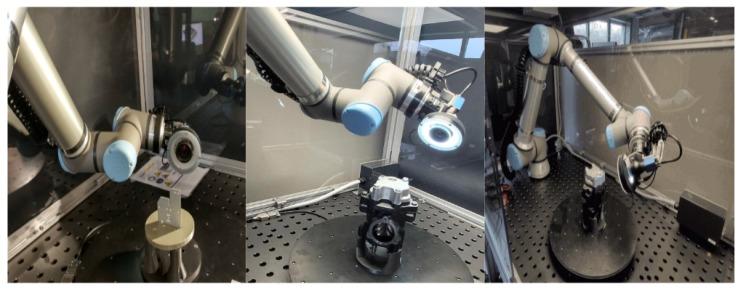
The active vision setup (AVS) with robot, camera, objects and lights.

Our AI-driven defect detection pipeline begins with a robust data collection process using our active vision setup (AVS), depicted in Figure 2. The system is equipped with a robotic arm that is mounted with a camera. The object under inspection, such as a metallic plate, is placed on a turntable, allowing the system to capture multiview images from different angles. To ensure optimal illumination, various light settings are integrated, with lights mounted around the camera and on the corners of the table. This entire setup is enclosed in a transparent glass box to maintain controlled lighting conditions and prevent interference from external factors.

The first step of the pipeline is data collection, where the AVS captures images from multiple angles using automated viewpoint optimization. This process is key to ensuring that the entire surface of the object is inspected and that subtle defects, such as scratches, are captured from various perspectives.

### 3.2. Viewpoint Optimization

In the current manufacturing landscape, the trend towards mass customization and high-quality demands poses unique challenges. Human-operated quality control offers flexibility but lacks consistency and repeatability. Automated systems, such as those employing robotic arms equipped with cameras, provide a promising solution. These systems must address key issues such as optimal viewpoint selection and efficient path planning to ensure comprehensive coverage of inspected surfaces.

To this end, we draw on techniques from Glorieux et al. [13], who proposed a coverage path planning method with targeted viewpoint sampling for robotic free-form surface inspection. Their approach optimizes viewpoint selection and path planning to minimize cycle times while ensuring thorough inspection coverage. Building upon these concepts, we adapt these techniques to our active vision setup for capturing multiview data. This ensures complete surface inspection, particularly for subtle defects such as scratches.

The exact algorithm behind our viewpoint optimization will be published separately. The focus of the current paper is on performing defect detection on the obtained figures, independently of how these viewpoints are selected (manually or automated) and independent of robot configuration parameters.

### 3.3. Auto-Annotation

**Figure 3:**
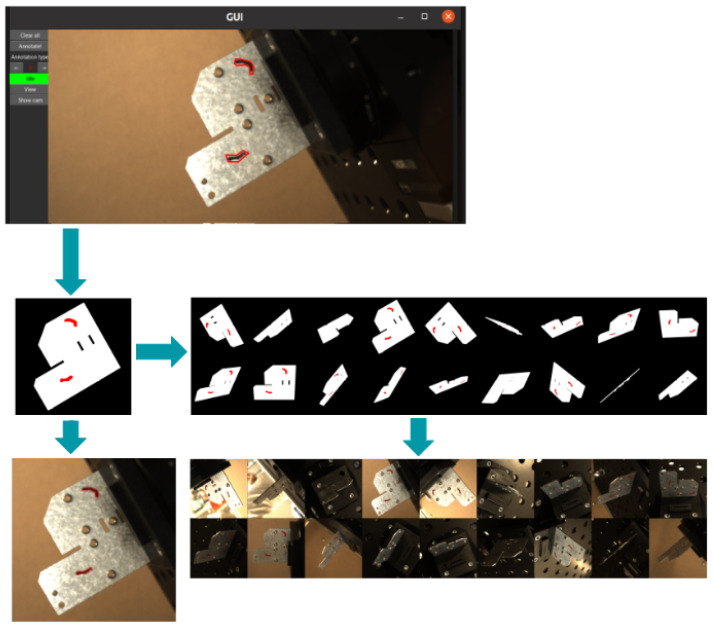
Examples of annotated images from the auto-annotation tool.

In our project, we set out to significantly reduce the manual effort required to generate large annotated machine vision datasets for surface defect detection. Our strategy is designed to utilize just a single manual annotation to generate annotations for several images of a defective object, aiming to streamline the process and increase efficiency substantially (Figure 3). This endeavor is driven by the recognition that deep learning neural network models demand extensive datasets to achieve high accuracy. However, the annotation of such data is labor intensive and often necessitates expertise, quickly becoming a bottleneck in the workflow. By leveraging an active vision system, which provides abundant information, including 3D data from computer-aided design (CAD) models and the camera’s six degrees of freedom in pose, we can alleviate the annotation workload.

Our approach employs a robot to capture images from various viewpoints of an object. Utilizing the robot’s forward kinematics and a calibration process, we accurately calculate the object’s 3D pose relative to the camera frame. The auto-annotation tool allows a user to select and annotate a single image. Upon completion, a segmentation mask is generated from this annotation. This mask is used to project a 3D ray from the annotated pixel through the camera’s lens, using its pose and intrinsic calibration, toward the CAD model. At the intersection point on the model, a dot is marked on the CAD’s texture map, indicating the defect’s location. Using this enriched texture map, we then employ a standard renderer to generate segmentation maps for each image in the dataset.

The results of our approach are promising, enabling the generation of datasets with significantly reduced manual annotation effort. A single manual annotation can be amplified to produce annotations for up to twenty images, dramatically cutting down on the time required for annotation. This efficiency not only facilitates the rapid collection of new datasets but also enhances the capability to learn and detect new surface defects, marking a considerable advancement in the field of machine vision.

While the details of the auto-annotation tool are foundational to this framework, additional advancements and comprehensive evaluations of the tool will be presented in a future publication. In this work, we focus on its application and contribution to the dataset preparation for defect detection.

### 3.4. Data Preprocessing

#### 3.4.1. Preprocessing

**Figure 4:**
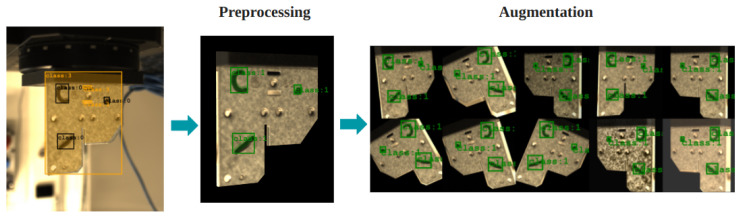
Preprocessing techniques: data preprocessing and augmentation process.

In active vision AI, preprocessing shapes raw data into a clean format for better model learning. We start by removing the background, making objects stand out for the AI to focus on. This step also simplifies what the AI sees, removing unnecessary details. Next, we crop images to focus on the most important parts, or the Region of Interest (ROI). This makes the AI pay attention to the key areas, using its resources more effectively. Then, we resize images to make them a standard size, helping the AI process them faster. This does not mean that we lose important details; it is about finding the right balance for efficiency and clarity. These steps, from removing backgrounds to resizing, are all about making the data as clean and straightforward as possible (Figure 4). This way, the AI can learn from the best possible examples, making it smarter and more accurate in understanding and interpreting visual information.

#### 3.4.2. Augmentation

After preprocessing, we move on to data augmentation, an essential step to improve our AI’s robustness and adaptability. We utilize the Albumentations library, which allows us to transform images along with their annotations easily. This includes a variety of random augmentations to expand our dataset artificially and make our model more resilient to different visual scenarios. One basic augmentation is the horizontal flip, which mirrors images across the vertical axis. This simulates the variability in object orientation that the AI might encounter. We also introduce rotations of up to certain degrees based on the data to mimic the tilt and angle changes in real-world observations, enhancing the model’s ability to recognize objects from various perspectives. Adjustments in color, brightness, and contrast are also applied randomly. These changes help the model learn to identify objects under diverse lighting conditions and color variations, ensuring consistent performance regardless of the visual environment. In addition to these transformations, we incorporate randomness in the preprocessing stage. For example, we intentionally introduce slight errors in background removal. This might seem counterintuitive, but it is designed to train the model to be forgiving of imperfections and to focus on the essential features of an object.

These preprocessing and augmentation steps ensure that the defect detection networks operate on clean, consistent, and diverse datasets, improving their robustness and accuracy.

### 3.5. Defect Detection Networks

#### 3.5.1. Semantic Segmentation

The decision to use semantic segmentation stems from its ability to handle defects that are elongated, irregularly shaped, or vary significantly in size. Unlike box-based methods, which are limited to concise or fixed-shape defects, semantic segmentation excels in detecting and localizing irregular defects by classifying each pixel. This makes it particularly effective for challenges such as detecting scratches on metallic plates, where defect shapes are often complex and subtle.

For semantic segmentation, we utilize the Segmentation Models PyTorch library by Iakubovskii [14] (version 0.4.0), a well-established framework offering pre-implemented state-of-the-art models. From this library, we evaluate 3 popular segmentation methods: UNet [15], FPN [5] and Deeplabv3+ [16]. To all three methods, we apply the same ResNet-34 backbone, as it strikes a good balance between speed and accuracy.

UNet [15] is a widely used fully convolutional network (FCN) designed specifically for medical image segmentation but has since been adopted for various semantic segmentation tasks, including defect segmentation. It follows a symmetric encoder–decoder structure, where the encoder captures hierarchical features through successive downsampling, while the decoder progressively upsamples the feature maps to restore spatial resolution. A key aspect of UNet is its skip connections, which directly transfer low-level features from the encoder to the decoder, preserving fine-grained spatial information and improving segmentation accuracy.

FPN [5] enhances feature representation by leveraging a top–down and lateral connection approach. Unlike traditional architectures that rely solely on the deepest feature maps, FPN constructs a multi-scale feature pyramid that integrates both high-level semantic information and fine-grained spatial details. This structure allows for better segmentation of objects at varying scales, making it particularly effective for complex and multi-scale scene parsing.

DeepLabv3+ [16] extends the DeepLab family by incorporating atrous (dilated) convolutions and an improved decoder module. Atrous convolutions enable a larger receptive field without increasing computational complexity, allowing the network to capture contextual information effectively. The addition of an explicit decoder further refines the segmentation results by recovering the spatial details lost during downsampling. DeepLabv3+ is known for its robustness in segmenting objects with complex boundaries and varying sizes.

This comparison serves as a benchmark to find the best base network to investigate different fusion methods.

#### 3.5.2. Early and Late Fusion

In the field of computer vision, especially within applications that require multiview analysis such as defect detection, the integration of information from multiple perspectives is crucial for enhancing the accuracy and robustness of detection algorithms. Two primary methodologies for the integration of multiview information are known as late fusion and early fusion. Both approaches offer distinct advantages and operational mechanisms in combining data from multiple sources to improve the overall decision-making process of neural networks.

**Figure 5:**
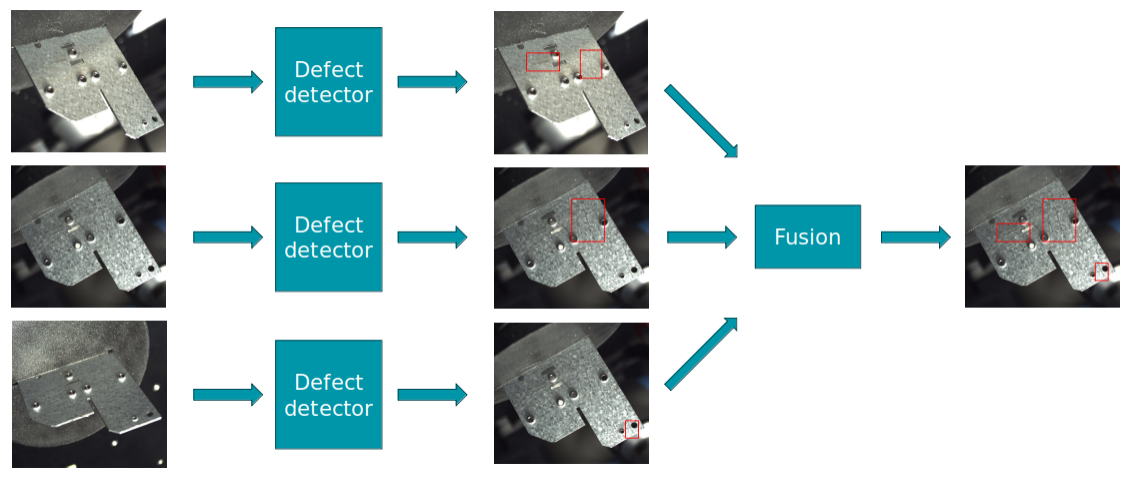
The late fusion architecture. Each image is processed independently by the defect detector, after which all detections are fused together. The red boxes indicate detected defects.

Late fusion refers to the technique where individual data views are processed independently through parallel pipelines, and their outputs are combined or fused towards the end of the process. This approach allows for specialized processing of each view, taking advantage of unique features or characteristics before making a collective decision. The primary advantage of late fusion lies in its simplicity and the flexibility it offers in processing and integrating heterogeneous data sources. The architecture of late fusion is illustrated in Figure 5, which demonstrates the separate processing pipelines for each view and their fusion at the decision level.

**Figure 6:**
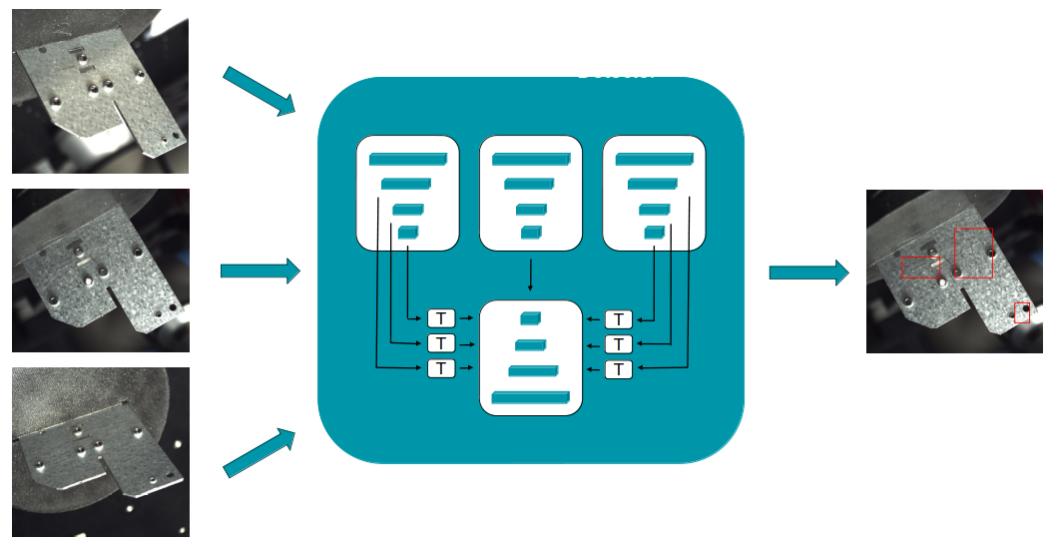
The early fusion architecture. The images (viewpoints) are processed by the defect detector at the same time, allowing the model to gather evidence for possible defects from different viewpoints. The red boxes indicate detected defects.

Early fusion, on the other hand, involves the integration of data from different views at an early stage, before or during the initial processing layers of the network. By feeding the network with a composite view that combines multiple perspectives, early fusion enables the model to leverage the interrelationships between different data views from the beginning. This approach is hypothesized to facilitate a more holistic understanding of the data, potentially leading to more accurate and robust detections by allowing the network to reason about the data in a more unified manner. Figure 6 provides an overview of the early fusion architecture, where data from multiple views are combined at the feature level before subsequent processing.

Our research delves into the comparative efficacy of these fusion methodologies within the multiview defect detection domain. We speculate that early fusion’s capability for concurrent multiview consideration could lead to more reliable detection results. To actualize early fusion, we introduce a novel architecture named MV-UNet (Multiview UNet), specifically crafted to align deep features from varied views.

The MV-UNet architecture modifies the UNet architecture to be able to reason about multiview inputs. The MV-UNet architecture consists out of N (with N the number of views) UNet encoders and a single UNet decoder. Whereas in the traditional UNet architecture the features of the encoder block are simply merged (concatenated) with the features of the decoder block, in our case, it is not that straightforward, as we are dealing with multiview features that are not aligned. To solve this issue, we incorporate a new merging block (a T-block), which transforms and merges the multiview features of the N encoders, after which they are concatenated with the decoder features. This T-block consists out of a perspective transformation module, performing a perspective transformation (homography) onto the features, aligning them to a reference view. In our case, we align the features to one of the multiview inputs. Given that we have a fully calibrated setup, we can easily calculate the required parameters for the T-blocks, as we know each camera’s location with respect to the object.

In contrast to early fusion, the late fusion approach leverages separate processing channels for each view, integrating the detection outcomes only in the final stages through a sophisticated fusion mechanism. By comparing both methodologies, our aim is to uncover insights into how multiview information can be most effectively leveraged for defect detection, contributing to the development of more sophisticated and reliable computer vision systems.

### 3.6. Evaluation Metrics

One important aspect of evaluating deep learning methods for industrial applications such as defect detection is the careful selection of evaluation metrics that reflect the model’s performance while being relevant to the specific use case. For scratch detection, widely used detection metrics such as precision, recall, and their combined metric, the F1-score, are often preferred over more complex metrics like mAP because they are simpler to understand and can be directly tied to quality standards in industry.

Precision measures how many of the detected defects are actual defects, while recall evaluates how many of the actual defects were correctly detected by the model. These metrics are expressed mathematically as

$$\mathrm{Precision}=\frac{\mathrm{True}\;\mathrm{Positives}}{\mathrm{True}\;\mathrm{Positives}+\mathrm{False}\;\mathrm{Positives}}$$

$$\mathrm{Recall}=\frac{\mathrm{True}\;\mathrm{Positives}}{\mathrm{True}\;\mathrm{Positives}+\mathrm{False}\;\mathrm{Negatives}}$$

The F1-score, is the harmonic mean of precision and recall, balancing the trade-off between the two:

$$F1=2\cdot \frac{\mathrm{Precision}\cdot \mathrm{Recall}}{\mathrm{Precision}+\mathrm{Recall}}$$

While these metrics are useful, they are not without limitations, especially in the context of scratch detection. One key challenge is the ambiguity that arises from ground-truth labeling and the strict rules used to match predictions with ground-truth instances. In detection-based methods, the IoU metric is often used to measure the overlap between predicted and ground-truth bounding boxes. However, this approach presents issues when the ground-truth label itself is ambiguous. For example, a single long scratch might be labeled as one defect, or it could be split into two smaller scratches, depending on how the annotator chooses to define the scratch. Similarly, if the model detects the scratch as two smaller instances, traditional precision and recall metrics would penalize this result, even though this distinction is often not meaningful in industrial contexts.

To overcome this challenge, it is frequently recommended—and it is also the approach used in this paper—to move away from detection-based methods and use segmentation methods instead. Segmentation inherently avoids some of the ambiguities associated with bounding-box labeling since it works at the pixel level. However, this shift raises a second issue: how can we maintain the traditional precision–recall metrics in the context of segmentation methods, which use pixel-based ground-truth masks rather than discrete instances?

**Figure 7:**
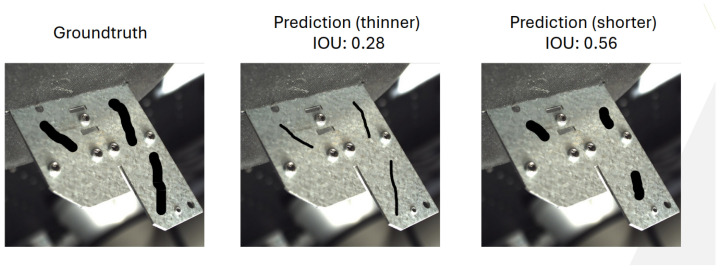
Intersection over Union (IoU) limitations for subtle defect detection: thin or short predictions achieve low IoU despite correctly capturing the defect.

The typical metric used for segmentation tasks is the IoU, which calculates the intersection-over-union between the predicted and ground-truth masks. This is an effective metric when dealing with segmentation at the pixel level, but it can be too strict for our use case. IoU penalizes discrepancies in the predicted mask’s shape or thickness compared to the ground-truth mask. In the case of scratch detection, small differences in the thickness or boundary of a scratch may be irrelevant for industrial applications but would still result in a low IoU score. The problem here is that the thickness of the scratch is often less important than whether the scratch itself is detected. For instance, a slightly thicker or thinner predicted mask compared to the ground-truth mask could still correctly identify the defect, but an IoU score would penalize this. See Figure 7.

This strictness of the IoU metric for segmentation is problematic in cases like scratch detection, where the exact boundary or thickness may not be as critical. To address this, we propose a modification of the traditional precision and recall metrics, adapted to the segmentation context. These metrics maintain the essence of the original precision and recall calculations but are better suited to the pixel-based nature of segmentation.

To calculate precision, we convert the binary prediction mask into individual components (by using a connected components algorithm), and for each component we evaluate their overlap with the binary ground-truth mask. The overlap is computed as the ratio of the intersection between the component and ground truth to the total component’s area. If the overlap exceeds a predefined threshold (e.g., 50%), the instance is considered correctly predicted. Precision is then calculated as the ratio of correctly predicted components to the total number of components:

$$\mathrm{Precision}=\frac{\mathrm{Correct}\;\mathrm{Components}}{\mathrm{Total}\;\mathrm{Components}}$$

For recall, we convert the binary ground-truth mask into individual components and assess their overlap with the binary prediction mask. The overlap is computed as the ratio of the intersection between the binary prediction mask and ground truth component to the total ground-truth component’s area. If the overlap exceeds a predefined threshold (e.g., 50%), the instance is considered correctly detected. Recall is calculated as the ratio of correctly detected ground-truth components to the total number of ground-truth components:

$$\mathrm{Recall}=\frac{\mathrm{Correctly}\;\mathrm{Detected}\;\mathrm{Components}}{\mathrm{Total}\;\mathrm{Ground}\;\mathrm{Truth}\;\mathrm{Components}}$$

Finally, the F1-score is derived from the precision and recall as

$$F1=2\cdot \frac{\mathrm{Precision}\cdot \mathrm{Recall}}{\mathrm{Precision}+\mathrm{Recall}}$$

This modification ensures that the evaluation metrics focus on meaningful overlap and detection accuracy, while avoiding the unnecessary penalization caused by small discrepancies in the thickness or boundary of the scratch. By adapting the traditional precision–recall metrics for segmentation, we can effectively evaluate models on scratch detection tasks without being overly sensitive to pixel-level variations.

These metrics, adapted to the segmentation task, offer a more reliable and industry-relevant evaluation of scratch detection models, accounting for practical requirements without being unduly affected by small variations in thickness or shape.

### 3.7. Results

This section assesses the performance of the proposed defect detection framework on a metallic plates dataset. The dataset comprises six distinct metallic plates, each captured in 64 images—32 of the front side and 32 of the back side. It is partitioned into an 80-20 train–evaluation split to ensure robust model assessment. The dataset presents typical challenges encountered in industrial quality control, particularly the detection of subtle surface scratches. The primary objective of this evaluation is to validate the effectiveness of the proposed early fusion and late fusion networks in comparison to single-view semantic segmentation for defect detection.

#### Experimental Setup

All networks—encompassing the (mono-view) semantic segmentation models and fusion networks—are trained until convergence using the binary cross-entropy loss function. The learning rate is set at $5\times 10^{-4}$ combined with the AdamW optimzer. Each dataset is partitioned into 80% training and 20% testing subsets to ensure a comprehensive evaluation. The performance of the models is assessed using precision, recall, and F1-score, which are tailored to defect detection tasks as described earlier.

The time required for capturing multiview images depends on factors such as the number of viewpoints, camera movement speed, and processing efficiency. Our active vision system is optimized with pre-calibrated viewpoints to reduce repositioning and improve scanning speed. For a typical metallic surface, the scanning process takes less than a minute per object. While multiview acquisition introduces a slight overhead compared to single-view methods, the gain in defect detection accuracy justifies this trade-off. Additionally, the early fusion network is designed to work with a minimal number of viewpoints, ensuring a balance between accuracy and inference time. The total data processing time is highly dependent on the number of viewpoints and compute infrastructure.

We first compare the score of 3 different segmentation models on our dataset. The results are shown in Table 1. We see that the DeeplabV3+ model performs best on the mono segmentation task, followed closely by UNet and FPN. Although the Deeplab model performs best, we decide to continue our fusion experiments with the UNet model, as it strikes a good balance between network complexity and accuracy.

The performance comparison of mono-view semantic segmentation (UNet), early fusion (MV-UNet), and late fusion networks (UNet) is presented in Table 2. The experiments highlight the following:

1. Mono-View Baseline: While the mono-view approach achieves a high precision of 0.928, its recall is limited to 0.875, leading to an F1-score of 0.901. This indicates that the single-view method struggles to comprehensively capture all defects due to its restricted perspective.
2. Late Fusion: The late fusion network shows a significant improvement in recall (0.990) compared to mono-view, demonstrating its ability to aggregate multiview predictions effectively. However, the precision decreases slightly (0.888), resulting in an F1-score of 0.936.
3. Early Fusion: The early fusion network achieves the highest overall F1-score (0.942), with a strong balance between precision (0.944) and recall (0.940). This indicates that early fusion is particularly effective in integrating multiview data at the feature level, providing robust detection results.

The following are the key observations from the results:

1. Holistic Inspection: Robust defect detection requires a holistic view of the object, inspecting it from multiple angles. Both early and late fusion approaches significantly outperform the mono-view method.
2. Fusion Network Comparison: Late fusion excels in recall, making it suitable for applications where maximizing defect coverage is critical. Early fusion achieves the highest precision and F1-score, indicating its ability to provide a balanced and robust defect detection performance.
3. Challenges in Early Fusion: While early fusion achieves superior results, it is computationally more challenging due to the need for precise feature alignment across multiple views.

**Figure 8:**
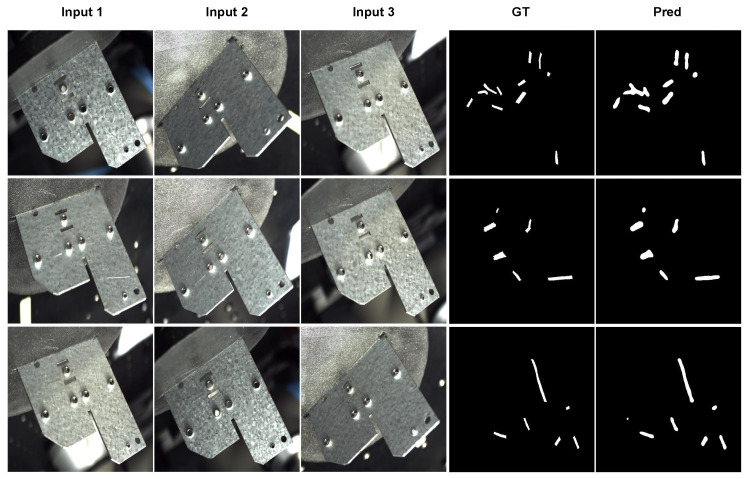
Early fusion results on the plates dataset. The MV-UNet takes the 3 views as input (Input 1, Input 2 and Input 3) and outputs a segmentation map aligned with Input 1, segmenting all scratches. The first three columns are the input to the MV-UNet, the fourth column is the groundtruth with respect to Input 1, and the last column is the output/prediction.

Visual results for the early fusion approach are showcased in Figure 8.

## 4. Conclusions

This research demonstrates the potential of integrating multiview deep learning techniques to improve defect detection accuracy in industrial settings. By leveraging early and late fusion methodologies, we show that multiview analysis outperforms traditional mono-view approaches in detecting subtle defects, such as scratches, on complex surfaces. The MV-UNet architecture, designed for early fusion, proves particularly effective, achieving the highest F1-score by aligning features from multiple views, leading to more robust and comprehensive defect segmentation.

In addition to the advanced fusion techniques, we develop an active vision setup that automatically adjusts the viewpoint for optimal defect detection. This setup, coupled with our automated annotation workflow, significantly reduces manual labor, ensuring the rapid creation of high-quality training datasets. These innovations streamline the model training process, making it more efficient and scalable for industrial applications.

We also introduce adapted precision–recall metrics that are specifically tailored for segmentation tasks, addressing the common limitations of traditional detection metrics like IoU, especially in the context of fine-grained defects. This adjustment ensures that small discrepancies in the defect boundary or thickness do not negatively impact the performance evaluation, providing a more reliable assessment of the model’s capabilities.

The results of our experiments on metallic plates, along with the planned expansion to other defect types, underscore the versatility and robustness of our approach. By integrating multiview predictions and enhancing the model with real-time feedback from an active vision system, our framework is poised to significantly improve defect detection in industrial environments. Future work will focus on applying this framework to additional defect categories, such as dents and cracks, and optimizing it for deployment in dynamic, real-world manufacturing settings. Additionally, efforts will be directed toward refining dynamic viewpoint optimization and further reducing environmental noise and hardware limitations to ensure that these systems are scalable and effective across various industrial applications.

## Figures and Tables

**Table 1:** Comparison of different segmentation models for scratch segmentation.

Model	Precision	Recall	F1 Score
UNet (ResNet18)	0.928	0.875	0.901
FPN (ResNet18)	0.85	0.85	0.85
DeeplabV3+	0.92	0.91	0.91

**Table 2:** Performance comparison of semantic segmentation (mono-view), early fusion, and late fusion networks for detecting scratches on plates. Best scores indicated in bold.

Method	Plates		
	Precision	Recall	F1-Score
Mono-View	0.928	0.875	0.901
Late Fusion	0.888	**0.990**	0.936
Early Fusion	**0.944**	0.940	**0.942**

## Data Availability

Data are contained within the article.

## Abbreviations

The following abbreviations are used in this manuscript:

AI	Artificial Intelligence
AVS	Active Vision Setup
CNNs	Convolutional Neural Networks
CAD	Computer-Aided Design
MAP	Mean Average Precision
IOU	Intersection Over Union
AVS	Active Vision Setup
ROI	Region of Interest
FCN	Fully Convolutional Network
FPN	Feature Pyramid Network
ResNet	Residual Neural Network
MV-UNet	Multiview UNet
